# Outcome prediction in hospitalized COVID-19 patients: Comparison of the performance of five severity scores

**DOI:** 10.3389/fmed.2023.1121465

**Published:** 2023-02-08

**Authors:** Hsin-Pei Chung, Yen-Hsiang Tang, Chun-Yen Chen, Chao-Hsien Chen, Wen-Kuei Chang, Kuan-Chih Kuo, Yen-Ting Chen, Jou-Chun Wu, Chang-Yi Lin, Chieh-Jen Wang

**Affiliations:** ^1^Division of Pulmonary, Department of Internal Medicine, MacKay Memorial Hospital, Taipei, Taiwan; ^2^Department of Critical Care Medicine, MacKay Memorial Hospital, Taipei, Taiwan; ^3^Department of Medicine, MacKay Medical College, New Taipei City, Taiwan; ^4^Division of Cardiology, Department of Internal Medicine, MacKay Memorial Hospital, Taipei, Taiwan

**Keywords:** COVID-19, SEIMC score, mortality prediction, IRS-NLR score, VICE score

## Abstract

**Background:**

The aim of our study was to externally validate the predictive capability of five developed coronavirus disease 2019 (COVID-19)-specific prognostic tools, including the COVID-19 Spanish Society of Infectious Diseases and Clinical Microbiology (SEIMC), Shang COVID severity score, COVID-intubation risk score-neutrophil/lymphocyte ratio (IRS-NLR), inflammation-based score, and ventilation in COVID estimator (VICE) score.

**Methods:**

The medical records of all patients hospitalized for a laboratory-confirmed COVID-19 diagnosis between May 2021 and June 2021 were retrospectively analyzed. Data were extracted within the first 24 h of admission, and five different scores were calculated. The primary and secondary outcomes were 30-day mortality and mechanical ventilation, respectively.

**Results:**

A total of 285 patients were enrolled in our cohort. Sixty-five patients (22.8%) were intubated with ventilator support, and the 30-day mortality rate was 8.8%. The Shang COVID severity score had the highest numerical area under the receiver operator characteristic (AUC-ROC) (AUC 0.836) curve to predict 30-day mortality, followed by the SEIMC score (AUC 0.807) and VICE score (AUC 0.804). For intubation, both the VICE and COVID-IRS-NLR scores had the highest AUC (AUC 0.82) compared to the inflammation-based score (AUC 0.69). The 30-day mortality increased steadily according to higher Shang COVID severity scores and SEIMC scores. The intubation rate exceeded 50% in the patients stratified by higher VICE scores and COVID-IRS-NLR score quintiles.

**Conclusion:**

The discriminative performances of the SEIMC score and Shang COVID severity score are good for predicting the 30-day mortality of hospitalized COVID-19 patients. The COVID-IRS-NLR and VICE showed good performance for predicting invasive mechanical ventilation (IMV).

## 1. Introduction

In December 2019, there was an emerging viral infection outbreak in Wuhan, China. The pathogen was later identified as a new strain of coronavirus, severe acute respiratory syndrome coronavirus-2 (SARS-CoV-2), and the disease it caused was named coronavirus disease 2019 (COVID-19). The disease rapidly spread from Wuhan to the rest of the world (1).

The disease severity ranged widely, from asymptomatic or minor symptoms, such as rhinorrhea, productive cough, anosmia, ageusia, and fever, to more severe conditions, such as pneumonia, acute respiratory failure, and even acute respiratory distress syndrome (ARDS). It can progress rapidly (2, 3) and advanced life support with intensive care, such as oxygen therapy, non-invasive ventilation (NIV), and invasive mechanical ventilation (IMV), may be warranted. Excessive demand on healthcare services overwhelmed healthcare systems worldwide (4–7). Administration triage for optimized patient care became essential.

Clinical evaluation alone may lead to misjudgment, under- or overestimation of disease severity and result in suboptimal medical treatment and admission to an inappropriate setting (8). Disease severity scores have been proposed since the early 1980s to help physician decision-making and predict outcomes. For instance, the CURB-65 (confusion, uremia, respiratory rate, BP, age ≥ 65 years) score and qSOFA (quick sepsis-related organ failure assessment) score are clinically relevant predictive tools for community-acquired pneumonia and sepsis, but their risk prediction performance in COVID-19 is not satisfactory (9, 10).

Several predictive scores had been published (11–20), but only a handful of them had ever been validated externally (10). The first wave of the COVID-19 pandemic in Taiwan occurred with a delay of several months in comparison with the first waves in other countries; nevertheless, the waves had similar viral characteristics. During the first wave, all COVID-19 patients had to be admitted to a hospital for quarantine according to the Taiwan Centers for Disease Control (CDC) regulation (21), regardless of the severity. Therefore, our cohort may be more representative of the spectrum of COVID-19 disease and be a good cohort to validate the accuracy of the previous predictive scores. The Spanish Society of Infectious Diseases and Clinical Microbiology (SEIMC) score (11) and the Shang COVID severity score (12) were developed to predict mortality, while the COVID-intubation risk score-neutrophil/lymphocyte ratio (IRS-NLR) score (13), inflammation-based risk score (14), and ventilation in COVID estimator (VICE) (22) score were designed to predict the need for IMV. All five predictive scores are useful since only clinical parameters and commonly available laboratory results were included, but the accuracy of these scores has been uncertain. Herein, the primary aim of the present study was to validate these severity scores and predictive models to predict mortality and the need for IMV.

## 2. Materials and methods

### 2.1. Study design and patient selection

We retrospectively studied all COVID-19 adult patients admitted for COVID-19 from 1 May 2021 to 30 June 2021, the first wave of COVID-19 infection in Taiwan with low COVID-19 vaccination coverage, to MacKay Memorial Hospital, a tertiary referral center in Taipei, Taiwan. All patients were confirmed to be diagnosed by a polymerase chain reaction using a nasopharyngeal sample. Patients who were under 20 years of age or identified as “do not intubate (DNI)” were excluded. The patients’ medical records and laboratory results were reviewed. Five different kinds of predictive scores were calculated, including the Shang COVID severity score (12), SEIMC score (11), COVID-IRS-NLR score (12), inflammation-based risk scoring system (14), and VICE score (22). The Institutional Review Board of MacKay Memorial Hospital approved this study with approval number 21MMHIS330e.

### 2.2. Outcome measurement

Our primary outcome was 30-day mortality. The secondary outcome was intubation with IMV support. Of note, non-IMV and high-flow nasal cannula were not included in the secondary outcome. The patients were followed until they expired or were discharged, depending on which developed first.

### 2.3. Definitions

The severity of COVID-19 scores was calculated by laboratory tests performed on or within 24 h of hospital admission. The patients were assessed for the presence of diabetes mellitus, coronary artery disease (CAD), and home statin medication history, and these factors were extracted from the electronic medical records. The estimated glomerular filtration rate (eGFR) was defined as the modification of diet in renal disease [Modification of diet in renal disease (MDRD) equation, which was 186 × (creatinine) (−1.154) × (age) (−0.203) for males and 186 × (creatinine) (−1.154) × (age) (−0.203) × 0.742 in females].

### 2.4. Statistical analysis

Categorical variables are presented as numbers (percentages). The frequencies of categorical variables were compared using the chi-squared test or Fisher’s exact test. Continuous variables are reported as the mean ± standard deviation (SD). The means of two continuous variables were compared by the independent samples *t*-test. We used a univariable logistic regression model to determine variables that would be included in our predictive risk score algorithms for mechanical ventilation needs and in-hospital death. In addition, variables with *p* < 0.05 were considered statistically significant and then were entered into a multivariate logistic regression model to determine independent predictors. We built receiver operating characteristic (ROC) curves to assess the predictive performance of all scores for the primary and secondary outcomes. We calculated pooled areas under the curve (AUCs) and 95% confidence intervals (CIs). The Hosmer–Lemeshow test was used to evaluate the goodness of fit for logistic regression models. For all tests, a two-sided *p*-value less than 0.05 was considered significant. Data were analyzed using SPSS software (version 22; IBM Corporation, Armonk, NY, USA).

## 3. Results

A total of 311 patients were enrolled in this study period and were followed until they were discharged from our hospital or died. A total of 26 patients who refused intubation during respiratory failure with DNI orders were excluded, leaving 285 patients for inclusion in the analysis.

### 3.1. Patient characteristics

The patient characteristics are shown in Table 1. The mean age was 61.5 ± 14.8 years, and 156 patients (54.7%) were male. Additionally, patients with the comorbidity of diabetes accounted for 29.8% of the cohort, and CAD patients accounted for 7.4% of our cohort. The lowest SpO_2_ level recorded within 24 h of admission was 93.7 ± 6.2%, and the SpO_2_/FiO_2_ ratio was 402.4 ± 106.2. A total of 65 patients (22.8%) were intubated with ventilator support, and 25 patients died with an 30-day mortality of 8.8%. Only 4.6% (13/285) of our cohort received one dose of a COVID-19 vaccine at the time of admission. The majority of our population were not vaccinated.

**TABLE 1:** Baseline characteristics and laboratory findings among survivors and non-survivors among hospitalized COVID-19 patients.

	All patients	Non-survivors at 30 days	Survivors at 30 days	*P*-value	Univariate	
	*N* = 285	*N* = 25	*N* = 260		Odds ratio (95% CI)	*P*-value
Age (years)	61.5 ± 14.8	71.7 ± 10.3	60.6 ± 14.9	<0.001	1.07 (1.03–1.11)	<0.001
Gender (male, %)	156 (54.7%)	16 (64.0%)	140 (53.8%)	0.40		
DM	85 (29.8%)	11 (44.0%)	74 (28.5%)	0.11		
CAD	21 (7.4%)	5 (20.0%)	16 (6.2%)	0.03	0.26 (0.09–0.79)	0.02
Statin use	29 (10.2%)	5 (20.0%)	24 (9.2%)	0.15		
Lowest SpO_2_ (%)	93.7 ± 6.2	88.6 ± 8.5	94.1 ± 5.7	0.004	0.92 (0.87–0.96)	0.001
eGFR (MDRD) (ml/min/1.73 m^2^)	76.8 ± 36.3	53.6 ± 60.3	79.0 ± 32.3	0.05		
BMI (kg/m^2^)	26.0 ± 4.9	25.1 ± 7.1	26.1 ± 4.6	0.52		
SEIMC_score	7.8 ± 5.2	12.5 ± 5.2	7.3 ± 4.9	<0.001	1.17 (1.09–1.25)	<0.001
IRS-NLR_score	2.7 ± 2.4	5.4 ± 3.2	2.5 ± 2.1	<0.001	1.51 (1.28–1.78)	<0.001
Inflammatory_score	2.8 ± 1.9	4.3 ± 1.4	2.6 ± 1.8	<0.001	1.63 (1.27–2.10)	<0.001
Shang_severity_score	2.2 ± 1.6	4.0 ± 1.3	2.0 ± 1.5	<0.001	2.40 (1.72–3.36)	<0.001
VICE_score	0.18 ± 0.23	0.46 ± 0.32	0.16 ± 0.20	<0.001	51.8 (12.1–221.1)	<0.001
Procalcitonin (ng/ml)	1.1 ± 7.7	8.0 ± 21.3	0.4 ± 4.3	0.10		
D-dimer (ng/ml)	1,459.3 ± 1,998.2	3,634.3 ± 3,273.2	1,245.2 ± 1,690.4	0.001	1.00033 (1.00019–1.00047)	<0.001
Platelet (μL)	202,722.3 ± 86,245.4	185,240.0 ± 68,694.5	204,423.0 ± 87,690.0	0.29		
White blood cell count (L)	7,657.2 ± 7,765.6	9,668.0 ± 6,835.2	7,462.4 ± 7,834.3	0.18		
Albumin (g/dl)	3.9 ± 0.6	3.4 ± 0.6	3.9 ± 0.5	<0.001	0.24 (0.11–0.52)	<0.001
CRP (mg/dl)	7.1 ± 6.8	13.3 ± 8.3	6.5 ± 6.4	<0.001	1.13 (1.07–1.19)	<0.001

DM, diabetes mellitus; CAD, coronary artery disease; eGFR, estimated glomerular filtration rate; MDRD, modification of diet in renal disease; BMI, body mass index; CRP, C-reactive protein, LDH, lactic dehydrogenase.

Clinical and laboratory parameters that were associated with 30-day mortality and the need for IMV were identified (Tables 1, 2). The factors on admission were consistently predictive of both mortality and a requirement for IMV, and these factors included age, lowest SpO_2_, D-dimer, albumin, and C-reactive protein (CRP) level. Comorbid CAD is a predictor of mortality only. The odds ratio (OR) of age in mortality was 1.07 (95% CI: 1.03–1.11) and 1.03 (95% CI: 1.01–1.05) in IMV requirement. The CRP level is the most predictive laboratory parameter in both mortality and IMV need, with ORs of 1.13 (95% CI: 1.07–1.19) and 1.14 (95% CI: 1.09–1.19), respectively.

**TABLE 2:** Baseline characteristics and laboratory findings with and without ventilator use with COVID-19 infection.

	Ventilation use	No ventilation use	*P*-value	Univariate	
	*N* = 65	*N* = 220		Odds ratio (95% CI)	*P*-value
Age (years)	65.8 ± 11.5	60.3 ± 15.4	0.002	1.03 (1.01–1.05)	0.009
Gender (male, %)	42 (64.6%)	114 (51.8%)	0.09		
DM	24 (36.9%)	61 (27.7%)	0.17		
CAD	7 (10.8%)	14 (6.4%)	0.28		
Statin use	6 (9.2%)	23 (10.5%)	1.00		
Lowest SpO_2_ (%)	90.0 ± 9.2	94.7 ± 4.4	<0.001	0.88 (0.83–0.93)	<0.001
eGFR (MDRD) (ml/min/1.73 m^2^)	67.9 ± 42.5	79.4 ± 34.0	0.03		
BMI (kg/m^2^)	25.0 ± 5.9	25.9 ± 4.5	0.92		
SEIMC_score	9.7 ± 3.9	7.2 ± 5.4	<0.001	1.09 (1.04–1.15)	0.001
IRS-NLR_score	5.0 ± 2.8	2.0 ± 1.7	<0.001	1.81 (1.53–2.13)	<0.001
Inflammatory_score	3.7 ± 1.7	2.5 ± 1.8	<0.001	1.45 (1.24–1.71)	<0.001
Shang_severity_score	3.2 ± 1.4	1.8 ± 1.5	<0.001	1.76 (1.44–2.15)	<0.001
VICE_score	0.40 ± 0.30	0.11 ± 0.15	<0.001	187.0 (45.1–766.1)	<0.001
Procalcitonin (ng/ml)	2.5 ± 12.5	0.7 ± 5.6	0.27		
D-dimer (ng/ml)	2,609.7 ± 3,084.2	1,116.9 ± 1,368.6	<0.001	1.00032 (1.00018–1.00046)	<0.001
Platelet (μL)	188,328.1 ± 60,221.6	206,948.2 ± 92,196.0	0.06		
White blood cell count (L)	8,384.4 ± 5,714.4	7,444.7 ± 8,268.7	0.40		
Albumin (g/dl)	3.6 ± 0.5	3.9 ± 0.6	<0.001	0.35 (0.20–0.61)	<0.001
CRP (mg/dl)	12.0 ± 7.8	5.6 ± 5.8	<0.001	1.14 (1.09–1.19)	<0.001

DM, diabetes mellitus; CAD, coronary artery disease; eGFR, estimated glomerular filtration rate; MDRD, modification of diet in renal disease; BMI, body mass index; CRP, C-reactive protein, LDH, lactic dehydrogenase.

### 3.2. Comparison of mortality and intubation rate by risk class

**FIGURE 1:**
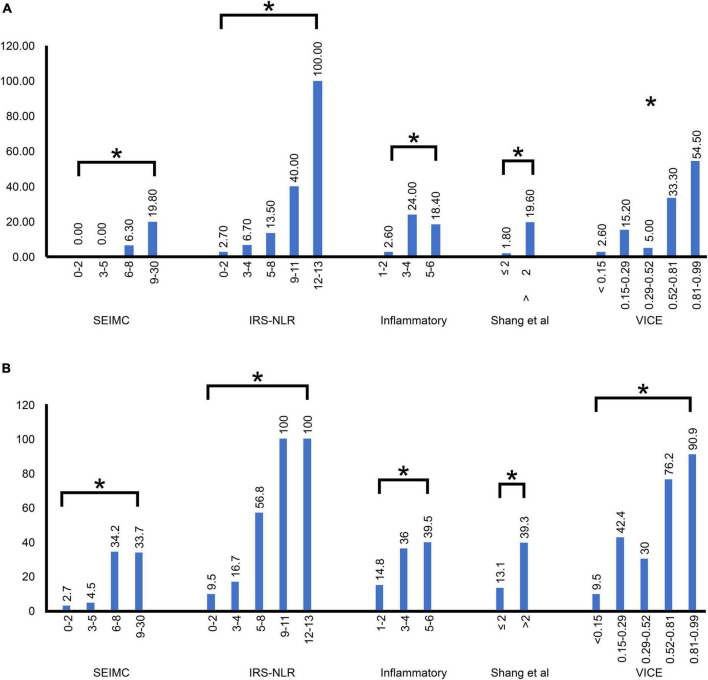
Distribution of the SEIMC, IRS-NLR, inflammatory, Shang et al., and VICE scores by risk class in our patients. **(A)** The mortality rate was plotted against five score stratifications. **(B)** The intubation rate was plotted against five score stratifications. The correlation between each of the five scoring systems and the increase in severity. *p < 0.001; SEIMC, Spanish Society of Infectious Diseases and Clinical Microbiology; IRS-NLR, intubation risk score-neutrophil/lymphocyte ratio; VICE, ventilation in COVID estimator.

Figure 1 shows the mortality rate and ventilation rate across different scoring system risk classes, including the COVID-19 SEIMC score, COVID-IRS-NLR score, inflammatory score, Shang COVID severity score, and VICE score. There was a significant difference in the mortality rate and intubation rate among the lowest- to highest-risk classes in all five scoring systems. The 30-day mortality increased steadily according to higher COVID-IRS-NLR, Shang COVID severity score, and SEIMC score. The intubation rate exceeded 50% in the patients stratified by higher VICE score and IRS-NLR score quintiles, which revealed 76.2 and 90.9% in the 4th and 5th quintiles in the VICE score and 56.8, 100, and 100% in the 3rd–5th quintiles in the IRS-NLR score, respectively.

### 3.3. Performance of risk prediction and modeling for COVID-19 mortality

**FIGURE 2:**
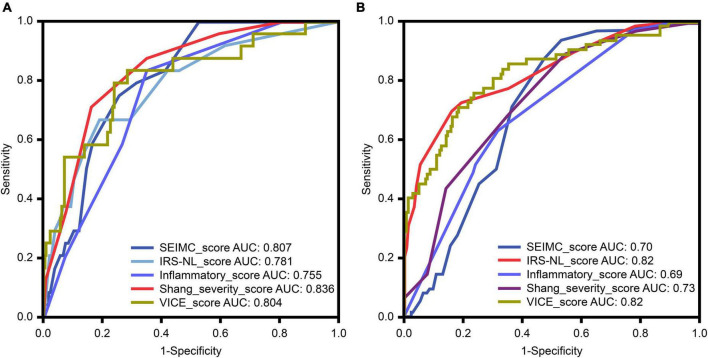
**(A)** The receiver-operator characteristic (ROC) curve (with AUC) for predicting mortality among patients with coronavirus disease 2019 (COVID-19) in our cohort. **(B)** The ROC curve for predicting mechanical ventilation requirements among patients with COVID-19 in our cohort. HR, hazard ratio; AUC, area under the curve; SEIMC, Spanish Society of Infectious Diseases and Clinical Microbiology; IRS-NLR, intubation risk score-neutrophil/lymphocyte ratio; VICE, ventilation in COVID estimator.

The area under the ROC curves (AUC) for 30-day mortality for each prognostic score for COVID-19 is shown in Figure 2A and Table 3. The Shang COVID severity score showed the highest prediction of mortality, with an AUC of 0.836. The AUCs for the SEIMC score and VICE score were 0.807 and 0.804, respectively, suggesting good predictive performance for 30-day mortality. The performance of the DICE score was not validated because of missing values in our cohort and loss of statistical power.

**TABLE 3:** Summary of the prognostic performance of different severity scores for mortality/intubation in hospitalized patients with COVID-19.

Risk score	Variables	Risk stratification in original study	OR (95% CI) for mortality, intubation*	RO-AUC for mortality, intubation*
Shang COVID severity score (12)	Age, coronary heart disease, Lymphocyte < 8%, Procalcitonin > 0.15 ng/ml, D-dimer > 500 ng/ml	Total score > 2 points defined as high risk; mortality rate 10% vs. 81.1% in low-risk group vs. high-risk group (*p* < 0.01)	2.40 (1.72–3.36), 1.76 (1.44–2.15)	0.836, 0.73
SEIMC (11)	Age, lowest SpO_2_, NLR, eGFR, dyspnea, sex	6–8 points defined as high risk, mortality rate was 10.6–19.5%; 9–30 points defined as very high, mortality rate was 27.7–100.0%	1.17 (1.09–1.25), 1.09 (1.04–1.15)	0.807, 0.70
Inflammation-based risk score (14)	WBC ≥ 9.3 × 10^3^ cells/μL, CRP level ≥ 13.0 mg/L, serum albumin level ≤ 3.6 g/dl	5–6 points defined as severe risk, 71% of IMV rate	1.63 (1.27–2.10), 1.45 (1.24–1.71)	0.775, 0.69
VICE (17)	DM, SpO_2_/FiO_2_, LDH	4th quintile defined as 0.52–0.81 points, 66.30% of IMV rate 5th quintile defined as 0.81–0.99 points, 90.20% of IMV rate	51.8 (12.1–221.1), 187.0 (45.1–766.1)	0.804, 0.82
COVID-IRS-NLR score (13)	Respiratory rate, SaFiO_2_, LDH, NLR	5–8 points defined as high risk, 36.6–69.5% of IMV rate 9–11 points defined as very high risk, 90.9–92.8% of IMV rate 12–13 points defined as very high risk, 100% of IMV rate.	1.51 (1.28–1.78), 1.81 (1.53–2.13)	0.781, 0.82

eGFR, estimated glomerular filtration rate; SpO_2_, peripheral arterial oxygen saturation; FiO_2_, fraction of inspired oxygen; SaFiO_2_, ratio of oxygen saturation to fraction of inspired oxygen; CRP, C-reactive protein; NLR, neutrophil to lymphocyte ratio; DM, diabetes mellitus; CRP, C-reactive protein; LDH, lactic dehydrogenase; IMV, invasive mechanical ventilation; OR, odds ratio; ROC-AUC, receiver-operator characteristic with area under the curve; CI, confidence interval.

*Results of external validation within our cohort.

Sex and other reliable mortality-associated variables, including age, lowest SpO_2_, CRP, albumin, and D-dimer, were selected. Logistic regression models were generated by combining the scoring systems with the above variables (Table 4). The risk predictive model with the VICE score, Shang COVID severity score, and IRS-NLR score showed significant prognostic accuracy for mortality [OR: 19.6; (3.06–126.0); 1.71 (1.09–2.69); 1.31 (1.06–1.62), respectively]. The Hosmer–Lemeshow test of all the models yielded a non-significant statistic, indicating that there was no departure from perfect fit.

**TABLE 4:** Models of predictors of mortality.

Models	Risk score	Odds ratio (95% CI)	*P*-value	Hosmer and Lemeshow test		
				*X* ^2^	df	*P*-value
Model 1	SEIMC_score	1.03 (0.85–1.25)	0.74	6.73	8	0.57
Model 2	IRS-NLR score	1.31 (1.06–1.62)	0.01	5.84	8	0.67
Model 3	Inflammatory_score	1.39 (0.90–2.13)	0.28	6.83	8	0.56
Model 4	Shang_severity_score	1.71 (1.09–2.69)	0.02	14.3	8	0.07
Model 5	VICE score	19.6 (3.06–126.0)	0.002	8.49	8	0.39

Model 1: age + sex + SpO2 at 24-h admission + C-reactive protein (CRP) + albumin + D-dimer + Spanish Society of Infectious Diseases and Clinical Microbiology (SEIMC) score.

Model 2: age + sex + SpO2 at 24-h admission + CRP + albumin + D-dimer + IRS-NLR_score.

Model 3: age + sex + SpO2 at 24-h admission + CRP + albumin + D-dimer + inflammatory score.

Model 4: age + gender + SpO2 at 24-h admission + CRP + albumin + D-dimer + Shang_severity_score.

Model 5: age + sex + SpO2 at 24-h admission + CRP + albumin + D-dimer + VICE_score.

### 3.4. Performance of risk prediction for intubation and further modeling

The ROC curves for the IMV requirement for each scoring system in COVID-19 patients are shown in Figure 2B and Table 3. The IRS-NLR and VICE scores showed the strongest prediction of mortality, with AUCs of 0.82 for both.

Logistic regression models were generated by combining scoring systems with sex, age, lowest SpO_2_, CRP, albumin, and D-dimer (Table 5). The risk predictive models with the VICE score and IRS-NLR score showed the greatest prognostic accuracy of intubation [OR: 84.9 (14.6–492.4) and 1.62 (1.33–1.99), respectively]. The Hosmer–Lemeshow test of all the models yielded a non-significant statistic, indicating that there was no departure from perfect fit.

**TABLE 5:** Models of predictors of ventilation use.

Models	Risk score	Odds ratio (95% CI)	*P*-value	Hosmer and Lemeshow test		
				*X* ^2^	df	*P*-value
Model 1	SEIMC_score	0.98 (0.86–1.12)	0.77	4.89	8	0.77
Model 2	IRS-NLR_score	1.62 (1.33–1.99)	<0.001	8.31	8	0.40
Model 3	Inflammatory_score	1.18 (0.88–1.58)	0.28	6.71	8	0.57
Model 4	Shang_severity_score	1.22 (0.90–1.66)	0.20	7.08	8	0.53
Model 5	VICE_score	84.9 (14.6–492.4)	<0.001	6.25	8	0.62

Model 1: age + sex + SpO2 at 24-h admission + C-reactive protein (CRP) + albumin + D-dimer + Spanish Society of Infectious Diseases and Clinical Microbiology (SEIMC) score.

Model 2: age + sex + SpO2 at 24-h admission + CRP + albumin + D-dimer + IRS-NLR_score.

Model 3: age + sex + SpO2 at 24-h admission + CRP + albumin + D-dimer + inflammatory score.

Model 4: age + gender + SpO2 at 24-h admission + CRP + albumin + D-dimer + Shang_severity_score.

Model 5: age + sex + SpO2 at 24-h admission + CRP + albumin + D-dimer + VICE.

## 4. Discussion

This study evaluated the performance of the COVID-19 SEIMC score, the COVID-IRS-NLR score, the inflammatory-based risk score, the Shang COVID severity score, and the VICE prediction rule in predicting 30-day mortality and IMV requirements in hospitalized COVID-19 patients. In our cohort, the SEIMC score and Shang COVID severity score were good models for predicting 30-day mortality. For intubation prediction, the IRS-NLR, and VICE score prediction rules showed the best performance.

Our results reinforce the results of several previous studies that found specific initial parameters to be significant predictors of poor outcome in patients with COVID-19. Age (23, 24), lower SpO_2_ (25), higher D-dimer, higher CRP, and hypoalbuminemia (26) increased the chances of mortality and ventilation requirements in our study. Previous studies found that an increase in D-dimer and fibrinogen is associated with an increase in COVID-19 severity and mortality (27–30). Increased D-dimer represents activation of coagulation cascades secondary to systemic inflammation and causes microthrombi formation inside the blood vessels that can induce disseminated intravascular coagulation (DIC) (31). CRP levels are reliable markers for prognostic factors (32). Taken together, these results indicate that COVID-19 virus-induced inflammatory and hypercoagulation responses drive the severity of disease (33).

Hundreds of prognostic scoring systems have been developed and studied during the COVID-19 pandemic to predict different outcomes, including mortality (11, 12, 15), severe illness (16–18), critical illness (34), intensive care unit (ICU) admission (19, 20, 35), or IMV use (13, 14). The definition of severe COVID-19 varies across different studies, and the hospitalization criteria may differ by disease prevention policy across countries. The complexity of the ICU admission criteria may fluctuate and be affected by demography and ICU bed scarcity (10). Therefore, the outcomes used in our study, 30-day mortality and IMV, are the most clinically relevant, while they have objective and reduced diversity.

Clinical scoring systems are designed to aid decision making and add to clinical judgment in different healthcare services. Some simplified scoring methods are designed to frequently assess dynamic requirements for escalating levels of respiratory support and to rescore after interventions, such as the Brescia-COVID respiratory severity scale (BCRSS) (36) and the Quick COVID-19 Severity Index (qCSI) (37). These kinds of scoring systems are practically applicable in emergent department settings, which allows quick triage. Most COVID-19 risk scores aimed to predict ultimate outcomes by using the initial evaluation, while some scores use parameters that have not been routinely collected in our cohort [such as CT scan (38) or red cell distribution width (39)]. Further prospective studies are needed for further validation.

To date, the largest, multicenter cohort of 14,343 patients to validate systematically selected prognostic scores for 30-day in-hospital mortality had been demonstrated that 4C mortality (15) and ABCS score had modest utility (AUC > 0.75) (10). The SEIMC scores had low prediction in that French cohort but good prediction in our cohort. The Shang et al. (12) severity score possesses the highest AUC-ROC curve in our patient population. The mortality rate observed within our cohort was much lower than expected in the Shang et al. (12) cohort. The mortality rate was 81.1% among the high-risk group (above 2 points), and the observed mortality rate of the high-risk group in our cohort was only 19.6%. The differences between the two cohorts may be because they faced the first wave of COVID-19, and there were no well-established treatment strategies. In contrast, the SEIMC score predicted the mortality rate in our cohort more precisely (11), while incremental risk stratification represented increased mortality.

The COVID-IRS-NLR and VICE score showed the strongest prediction and greatest accuracy of IMV among our patient population. The intubation rate of the high-risk and very high-risk patients based on the COVID-IRS-NLR score and those who were in quintiles 4th and 5th based on the VICE score was high (13, 22), and these factors may warrant intubation. Early intubation could prevent patient self-inflicted lung injury (40). However, IMV is also associated with complications, such as prolonged sedation and paralysis, infection, and barotrauma (41), and it was reasonable to treat the patients in the low-risk group using a high-flow nasal cannula or awake proning rather than early intubation.

Triage is important in the face of the COVID pandemic (42). If we can foresee the possible progress of the patient’s condition during the first episode, different approaches, treatment options, and patient relocation can be arranged accordingly (43, 44). Patients who are predicted to be less likely to worsen can be treated with a step-down approach and at home without consuming medical resources (45). Patients with a high risk of death or requiring mechanical ventilation support should be admitted to the hospital ward or even the ICU. Therefore, we can reduce the possibility of missed diagnosis of severe COVID-19 and mortality (43).

The underlying reasons for the performance differences among these severity scores could be multifactorial. Patient characteristics (46), vaccination status (47), healthcare system (48), and the variables included in each of the score all contribute to the performance differences. Table 3 shows the different variables used in each scoring systems. The variables could be categorized into patient characteristics, symptoms/signs, laboratory data, and clinical parameters, such as respiratory rate, SpO_2_/FiO_2_. Age was included both in the SEIMC score and Shang COVID severity score which were the better predictive scores of 30-day mortality. These finding implied the importance of age in driving severity among hospitalized COVID-19 patients (49). The good predictive scores for IMV in our study were the COVID-IRS-NLR and VICE score, and the common variables were lactic dehydrogenase (LDH) and SpO_2_/FiO_2_. Elevated LDH was associated with poor outcome in COVID-19 patients (50). SpO2/FiO2 may substitute for PaO2/FiO2 as a diagnostic and prognostic marker in COVID-19 patients (51). The performance differences do exist in different race/ethnicity and regions; external validation is needed (52). Multi-center, cross-country studies to verify the accuracy of these severity scores need to be conducted.

## 5. Limitations

This study had several limitations. First, it was conducted at a single center, and the results may not be generalizable worldwide. Second, the retrospective design with some missing values for several important variables, such as body mass index (BMI) and statin use, results in an inability to validate more scoring systems. Third, due to the small number of cases in our cohort, it may not be significant to identify new cut-off points in each score. Finally, our cohort was composed of COVID-19 alpha variant patients and most of them were unvaccinated. COVID-19 vaccination status is associated with the severity of COVID-19 illness. The prediction ability of the five risk scores for other variants or vaccinated populations is unknown. Further well-designed prospective studies are needed to validate these findings in the future.

## 6. Conclusion

The discriminative performance of the SEIMC score and Shang COVID severity score were good for 30-day mortality in COVID-19 hospitalized patients. For intubation prediction, the COVID-IRS-NLR, and VICE score prediction rules showed the best performance.

## Data availability statement

The raw data supporting the conclusions of this article will be made available by the authors, without undue reservation.

## Ethics statement

The studies involving human participants were reviewed and approved by the Institutional Review Board of MacKay Memorial Hospital. Written informed consent for participation was not required for this study in accordance with the national legislation and the institutional requirements.

## Author contributions

C-JW contributed to the conception and study design. H-PC and Y-HT were in charge of execution, acquisition of data, interpretation, and drafting. C-YC took charge of data analysis. C-HC, W-KC, K-CK, and Y-TC were in charge of execution, acquisition of data, analysis, and interpretation. J-CW and C-YL took charge of drafting, revising, and critically reviewing the article. All authors gave final approval of the version to be published, had agreed on the journal to which the article has been submitted, and agreed to be accountable for all aspects of the work.
